# Endothelial Galectin-1 Binds to Specific Glycans on Nipah Virus Fusion Protein and Inhibits Maturation, Mobility, and Function to Block Syncytia Formation

**DOI:** 10.1371/journal.ppat.1000993

**Published:** 2010-07-15

**Authors:** Omai B. Garner, Hector C. Aguilar, Jennifer A. Fulcher, Ernest L. Levroney, Rebecca Harrison, Lacey Wright, Lindsey R. Robinson, Vanessa Aspericueta, Maria Panico, Stuart M. Haslam, Howard R. Morris, Anne Dell, Benhur Lee, Linda G. Baum

**Affiliations:** 1 Department of Pathology and Laboratory Medicine, David Geffen School of Medicine at UCLA, Los Angeles, California, United States of America; 2 Department of Microbiology, Immunology, and Molecular Genetics, David Geffen School of Medicine at UCLA, Los Angeles, California, United States of America; 3 Division of Molecular Biosciences, Faculty of Natural Sciences, Imperial College, London, United Kingdom; 4 MSCAN Ltd., Millars Business Centre, Wokingham, Berks, United Kingdom; University of California Irvine, United States of America

## Abstract

Nipah virus targets human endothelial cells via NiV-F and NiV-G envelope glycoproteins, resulting in endothelial syncytia formation and vascular compromise. Endothelial cells respond to viral infection by releasing innate immune effectors, including galectins, which are secreted proteins that bind to specific glycan ligands on cell surface glycoproteins. We demonstrate that galectin-1 reduces NiV-F mediated fusion of endothelial cells, and that endogenous galectin-1 in endothelial cells is sufficient to inhibit syncytia formation. Galectin-1 regulates NiV-F mediated cell fusion at three distinct points, including retarding maturation of nascent NiV-F, reducing NiV-F lateral mobility on the plasma membrane, and directly inhibiting the conformational change in NiV-F required for triggering fusion. Characterization of the NiV-F N-glycome showed that the critical site for galectin-1 inhibition is rich in glycan structures known to bind galectin-1. These studies identify a unique set of mechanisms for regulating pathophysiology of NiV infection at the level of the target cell.

## Author Summary

Nipah virus (NiV) is classified as a “priority pathogen” by the NIH. NiV infection of humans results in multi-organ hemorrhage due to endothelial syncytia formation, and also causes fatal encephalitis in up to 70% of patients. As there are no effective vaccines or therapeutics for NiV, understanding the mechanism of endothelial damage by NiV is a critical goal. Our present work defines the interaction between galectin-1, an innate immune lectin that is secreted by human endothelial cells, with the fusion glycoprotein of NiV. We demonstrate that galectin-1 can block the function of the NiV-F protein via three distinct mechanisms, and thus reduce the ability of NiV-F to cause endothelial cell-cell fusion. Importantly, in this study, we use human endothelial cells, the primary target of Nipah virus *in vivo*, and demonstrate that endogenous galectin-1 made by endothelial cells contributes to limiting cell-cell fusion caused by NiV-F. As endothelial syncytia formation is one of the primary pathophysiologic events in Nipah virus infection, contributing to the hemorrhagic diathesis seen in infected patients, understanding the mechanism of endothelial cell fusion and the ability of galectin-1 to ameliorate cell fusion are critical for development of new approaches to mitigate these events.

## Introduction

Nipah virus (NiV) is a lethal emerging virus that infects agricultural livestock and humans. In 1999–2000, NiV infection of agricultural workers in Malaysia and Singapore resulted in a 40% mortality rate, and subsequent outbreaks in Bangladesh resulted in an average case-fatality ratio greater than 70% [1]. In humans, NiV targets endothelial and neural cells, with resulting respiratory and neurologic sequelae; patients infected with Nipah virus often succumb to acute encephalitis with accompanying multi-organ failure due to systemic vasculitis. Autopsy studies of NiV victims identified virus in endothelial cells, with endothelial cell syncytia formation being a pathognomonic hallmark of NiV infection [2], [3], [4].

NiV, a member of a new genus of *Paramyxoviridae*, is an enveloped virus with two viral envelope glycoproteins, NiV-G and NiV-F, that mediate viral entry [5]. The NiV-G attachment glycoprotein binds to specific receptors, primarily ephrinB2 or alternatively ephrinB3 [6], [7], [8]. While ephrinB2 and ephrinB3 are expressed in neuronal cells, only ephrinB2 is highly expressed on endothelial cells [9]. The NiV-F glycoprotein mediates fusion of bound virus with target cells. After endothelial cell infection, NiV-F and NiV-G glycoproteins are expressed on the surface of infected cells. This triggers cell-cell fusion with neighboring endothelial cells (homologous fusion) or stromal cells (heterologous fusion), resulting in endothelial syncytia formation, endothelial disruption, and subsequent hemorrhage and tissue damage.

The NiV-F fusion glycoprotein, NiV-F_0_, is initially expressed at the cell surface as a single glycosylated polypeptide precursor, but subsequently undergoes endocytosis and endosomal proteolytic cleavage by cathepsin L into F_1_ and F_2_ subunits that are disulfide linked to form the mature fusion protein NiV-F_1/2_ [10], [11], [12]. Mature NiV-F traffics back to the cell surface, where the protein can initiate cell-cell fusion at neutral pH when it is appropriately triggered by receptor binding to NiV-G. Our labs and others have previously demonstrated that specific N-glycans on NiV-F play important roles in regulating the extent of cell fusion [13], [14].

Infection of endothelial cells by viruses results in the release of innate immune effectors, including galectins, a family of mammalian lectins [15], [16]. Galectins are soluble, secreted proteins that stay associated with the cell surface by binding specific cell surface glycan ligands on specific glycoprotein receptors [17]. All galectins are multivalent or form higher order multimers, and can thus cross-link glycan ligands and the glycoprotein receptors that bear these ligands on the surface of cells. Human endothelial cells express galectin-1, as well as galectin-3, -8, and -9, and *in vitro* and *in vivo* activation of human endothelial cells increased synthesis as well as secretion and cell surface localization of galectin-1 [17], [18], [19].

We have previously shown that recombinant human galectin-1 inhibits cell fusion and syncytia formation caused by NiV-F [20]. The mechanism by which galectin-1 inhibits NiV-F mediated cell fusion is not well understood; however, we found that galectin-1 bound directly to NiV-F and caused NiV-F to oligomerize, suggesting that galectin-1 can cross link NiV-F on the surface of infected cells. Moreover, a single N-glycan site in NiV-F, the F3 glycosylation site, appears to be critical for galectin-1 mediated inhibition of cell fusion, as mutation of that site to abolish N-glycan addition, reduced galectin-1 binding to NiV-F and reduced the inhibitory effect of galectin-1 by 50% [20].

In the present study, we demonstrate that galectin-1 regulates NiV-F mediated fusion of endothelial cells and neural cells, the targets of NiV infection *in vivo*. Since endothelial cell syncytia formation is a pathognomic feature of NiV infection, we investigated the mechanisms involved in galectin-1 modulation of cell-cell fusion. We found that galectin-1 regulates cell fusion at three distinct points in the process; galectin-1 retains immature NiV-F_0_ on the cell surface to reduce production of the mature NiV-F fusion protein, galectin-1 reduces lateral movement of NiV-F on the plasma membrane that is required for cell-cell fusion, and galectin-1 directly inhibits the fusogenic activity of NiV-F by preventing fusion-peptide exposure and pre-hairpin intermediate (PHI) formation. The biological significance of our results is underscored by our demonstration that endogenous galectin-1 on endothelial cells is sufficient to reduce NiV-F mediated fusion. These studies identify a unique set of mechanisms for regulating the pathophysiology of NiV induced syncytia formation at the target cell level, and contribute to our understanding of the interaction between galectin-1 and glycoproteins of microbial pathogens.

## Results

### Cell surface galectin-1 inhibits syncytia formation in relevant cell types

**Figure 1:**
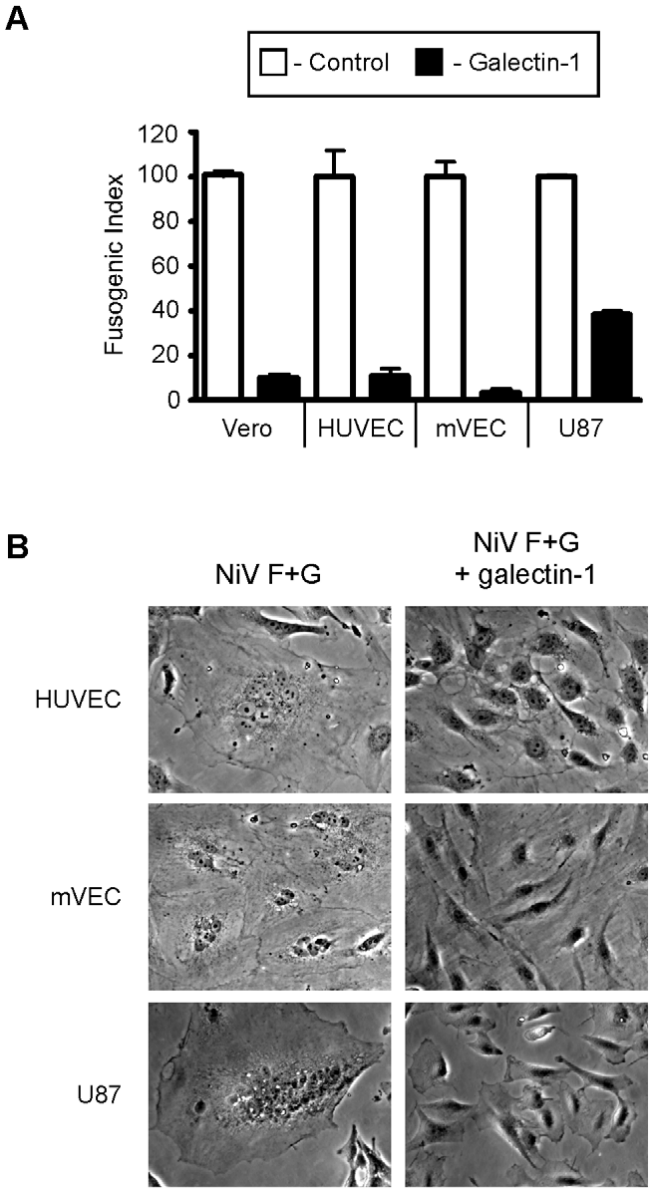
Galectin-1 blocks NiV-F and G mediated syncytia formation of endothelial and glial cells. **A**, Quantification of galectin-1 inhibition. PK-13 (ephrinB2 negative) cells expressing NiV-F and NiV-G were added to monolayers of ephrinB2 positive cells, Vero (control), HUVEC, mVEC, and U87. Heterologous fusion in the absence and presence of 20µM galectin-1 (white and black bars respectively) were quantified as described in Experimental Procedures. Data are mean ± SD of triplicate samples from one of three replicate experiments. **B**, Representative images of cell fusion in the absence or presence of galectin-1. Left panels show multinucleated syncytia in the indicated cell type. Right panels are cells treated with galectin-1 (20×).

We previously found that galectin-1 inhibits syncytia formation in Vero cells mediated by NiV-F and NiV-G [20]. *In vivo*, endothelial cells and neuronal cells are the main targets of Nipah virus [2], due to the high expression of ephrinB2 and/or ephrinB3 by these cells [6], [8]. To explore the role of galectin-1 in NiV-F and G mediated endothelial and glial cell syncytia formation, we used a heterologous overlay fusion assay. EphrinB2 and ephrinB3-negative cells (PK13) were transfected with NiV-F and NiV-G and plated on a confluent layer of the indicated target cell type (Fig. 1). Cell-cell fusion could be observed as early as 45 min and plateaued after approximately 6 hrs. Addition of recombinant galectin-1 treatment to the co-cultures significantly inhibited syncytia formation in each of the target cell lines. Vero cells, human umbilical endothelial cells (HUVECs), and microvascular endothelial cells (mVECs) all showed a 90% reduction in cell-cell fusion, and U87 glioblastoma cells showed a 60% reduction in fusion, compared to control cultures, in the presence of galectin-1 (Fig. 1A). Representative images from the syncytia assays for each target cell type are shown in Fig. 1B.

### Endogenous endothelial galectin-1 inhibits syncytia formation

**Figure 2:**
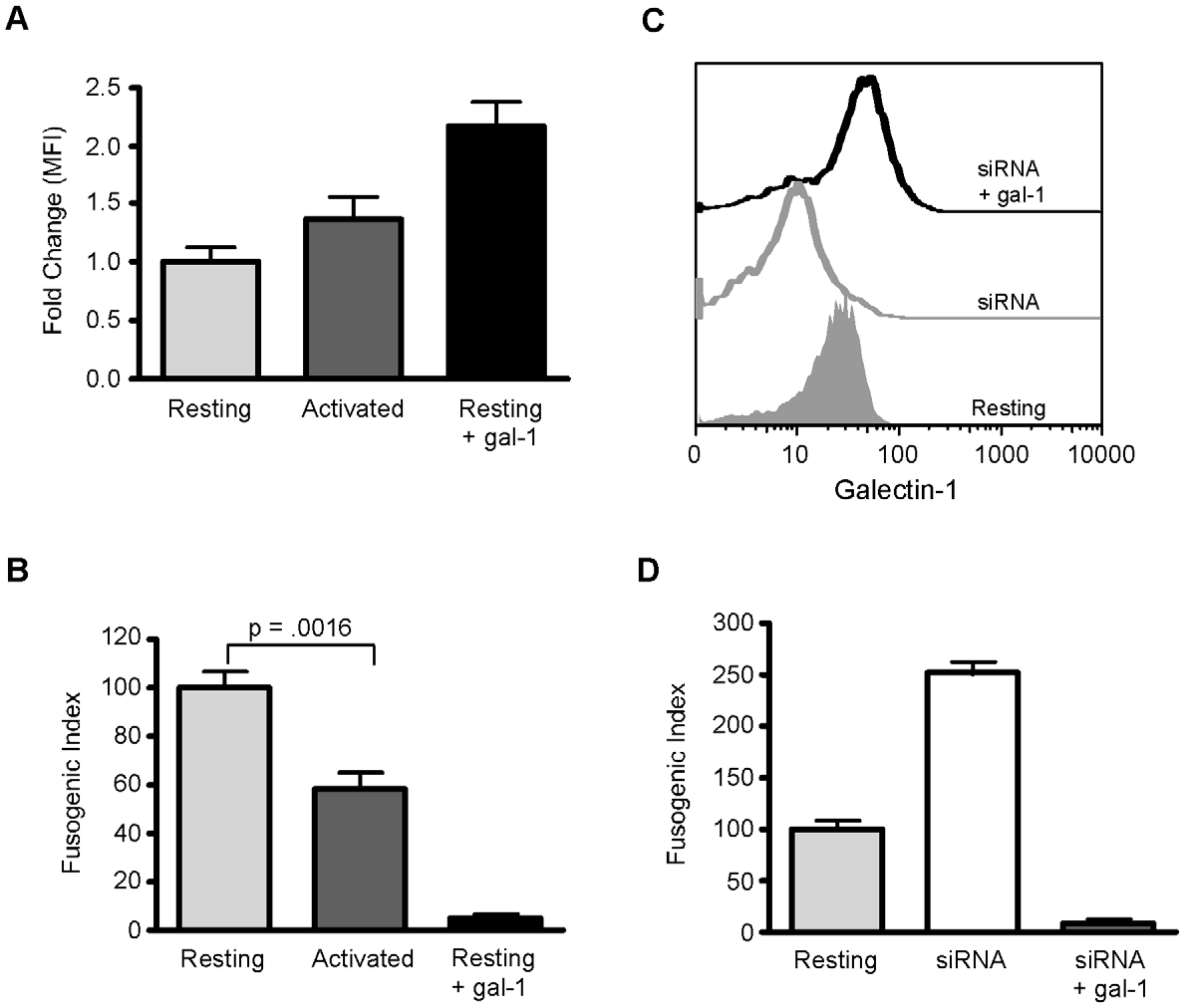
Endogenous endothelial galectin-1 inhibits NiV-F and NiV-G mediated syncytia formation. **A**, Activated HUVECs have increased cell surface galectin-1 compared to resting cells. Flow cytometric analysis of cell surface galectin-1 on resting (light grey) and activated (dark grey) HUVECs, and on resting HUVECs plus exogenous galectin-1 (black) Data are mean ± SEM of three independent experiments, each done in triplicate. **B**, Activated HUVECs are resistant to NiV-F and NiV-G mediated cell fusion. Heterologous cell fusion of resting (light grey) and activated (dark grey) HUVECs, and resting HUVECs plus exogenous galectin-1 (black). Data are mean ± SD of triplicate samples from one of three replicate experiments. **C**, Reduction of cell surface galectin-1 by siRNA. Flow cytometric analysis of cell surface galectin-1 on resting HUVECs (grey filled), siRNA treated HUVECs (grey line), and siRNA treated HUVECS with 20µM exogenous galectin-1 (black line). **D**, Reduction of cell surface galectin-1 in HUVECs increases susceptibility to NiV-F and G mediated cell fusion. Heterologous cell fusion of resting cells (light grey), cells with reduced galectin-1 (white), and cells with reduced galectin-1 plus exogenous galectin-1 (dark grey). Data are mean ± SD of triplicate samples from one of three replicate experiments.

Nipah virus infection results in extensive damage to endothelial cells as a result of syncytia formation, culminating in multi-organ hemorrhage and death. During viral infection, endothelial cells become activated and release immune mediators, including galectin-1 [18], [21]. We asked if endogenous endothelial galectin-1 could affect NiV-F mediated syncytia formation, using the heterologous fusion assay as in Fig. 1. To activate HUVECs, the cells were cultured in 20% human serum for four days, a process that increased cell surface galectin-1 protein expression in these cells [21]. Galectin-1 expression on the cell surface was quantified by flow cytometry, and activated HUVECs demonstrated a consistent increase in cell surface galectin-1 compared to resting cells (Fig. 2A). Activated HUVECs also showed a significant decrease (40–50%) in cell-cell fusion, which correlated with the increase in cell surface galectin-1 (Fig. 2B).

Conversely, in order to determine if endogenous galectin-1, even on resting HUVECs, was sufficient to affect NiV-F and G mediated syncytia formation, we reduced expression of galectin-1 in HUVECs using lentiviral vectors expressing siRNAs targeted against galectin-1. A combination of three siRNAs reduced galectin-1 protein approximately 70% (data not shown), and reduced cell surface galectin-1 approximately two-fold (Fig. 2C). Reduction of endogenous galectin-1 had a dramatic effect on syncytia formation, as HUVECs treated with siRNA to galectin-1 demonstrated 2.5-fold increase in syncytia formation, compared to cells treated with control siRNA (Fig. 2D). Infection of HUVECs with lentiviral vectors containing no siRNA, or siRNA against an irrelevant protein, had no effect on syncytia formation, which was identical to that observed in uninfected cells (data not shown).

To confirm that cell surface galectin-1 was responsible for the effect on syncytia formation, we added exogenous galectin-1 to HUVECs in which galectin-1 expression was decreased by siRNA. We observed the expected increase of cell surface galectin-1 (Fig. 2C), as well as a decrease in syncytia formation (Figure 2D). Thus, endogenous galectin-1 on the surface of endothelial cells inhibits NiV-F/G mediated syncytia formation, underscoring the biological relevance of the effect of galectin-1 on NiV mediated cell-cell fusion.

### Galectin-1 decreases the lateral mobility of NiV-F on the plasma membrane

**Figure 3:**
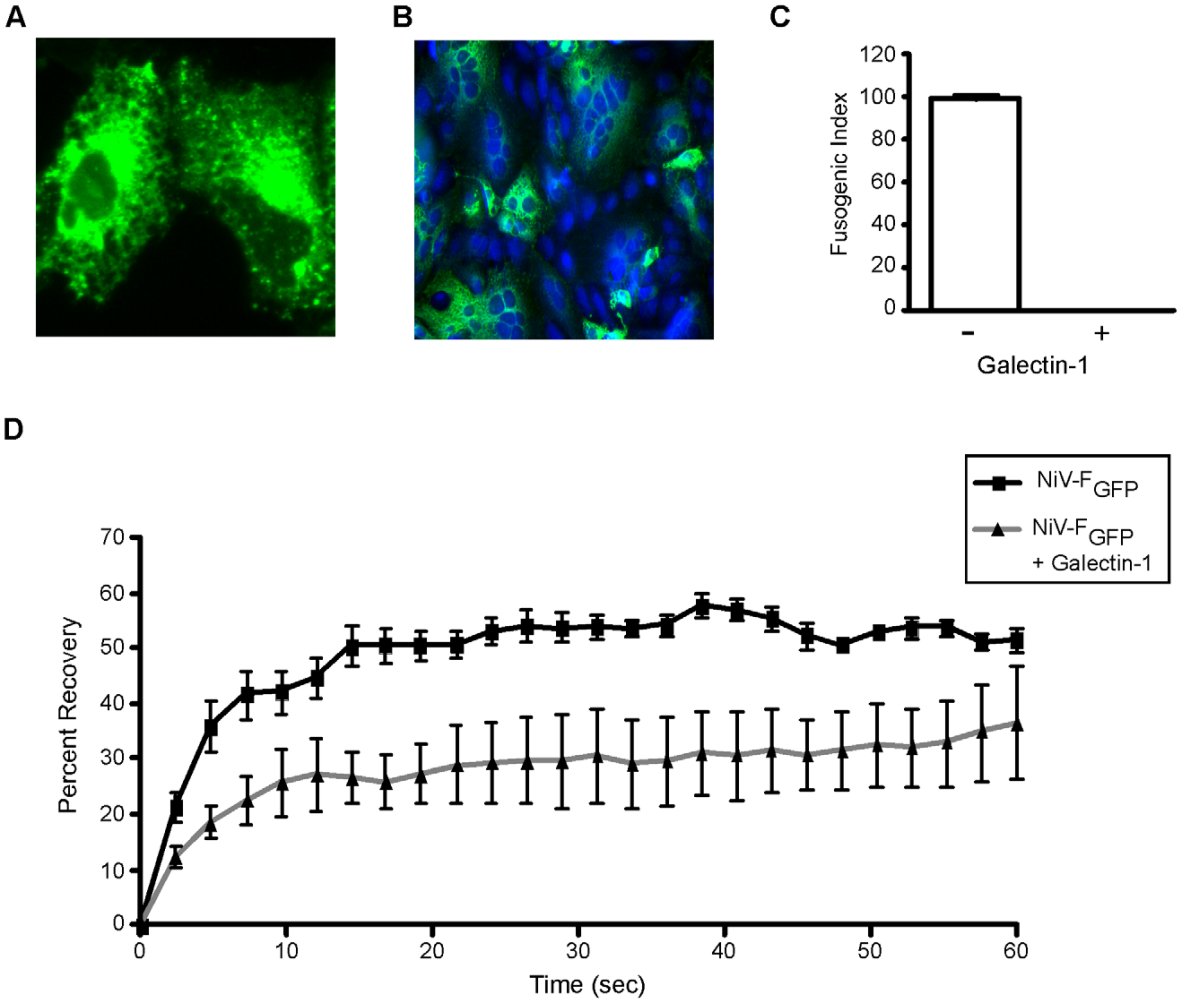
Galectin-1 interferes with lateral movement of NiV-F on the plasma membrane. **A**, NiV-F_GFP_ expression on the surface of two individual Vero cells. **B**, NiV-F_GFP_ promotes cell fusion when transfected into Vero cells with NiV-G. (20×) **C**, Galectin-1 inhibits fusion mediated by NiV-F_GFP_. Fusion of Vero cells in the absence (−) or presence (+) of 20µM galectin-1 was measured as in Fig. 1. **D**, Galectin-1 inhibited fluorescence recovery after photobleaching. NiV-F_GFP_ transfected Vero cells were treated with buffer control (black line with black squares) or 20µM galectin-1 (grey line with black triangles), and a portion of the membrane was bleached and measured for fluorescent recovery (y-axis) as a function of time in seconds (x-axis). Data are mean ± SD of six replicate measurements from one of two independent experiments.

Galectin-1 regulates the distribution and residence time of cell surface glycoproteins by binding to glycan branches on glycoproteins to create a cell surface lectin-glycoprotein lattice [22]. Lattice formation can have a variety of effects, including decreasing lateral mobility of glycoproteins. Lateral mobility is critical for effective cell-cell fusion mediated by NiV-F and NiV-G, as it is assumed that the F and G glycoproteins must physically separate in order to facilitate cell fusion [13], [23]. To determine the effect of galectin-1 on the lateral mobility of NiV-F, we performed fluorescence recovery after photobleaching (FRAP) analysis, using GFP-tagged NiV-F [24]. NiV- F_GFP_ was expressed on transfected Vero cells (Fig. 3A) and mediated cell-cell fusion when co-transfected with NiV-G (Fig. 3B). Galectin-1 was also efficiently able to inhibit fusion mediated by NiV-F_GFP_ and NiV-G (Fig. 3C).

To determine the effect of galectin-1 on NiV-F lateral movement, we measured the fluorescence recovery of NiV-F_GFP_ (lateral diffusion) in the presence or absence of galectin-1 (Fig. 3D). We observed 50–60% recovery of NiV-F_GFP_ fluorescence within 20 sec of photobleaching in the absence of galectin-1 (Fig. 3D). Addition of galectin-1 slowed the initial rate of fluorescence recovery, and also reduced overall fluorescence recovery to 25% (Fig. 3D). Thus, the presence of galectin-1 retarded NiV-F lateral movement on the cell surface.

### Galectin-1 prevents endocytosis of NiV-F and inhibits maturation

**Figure 4:**
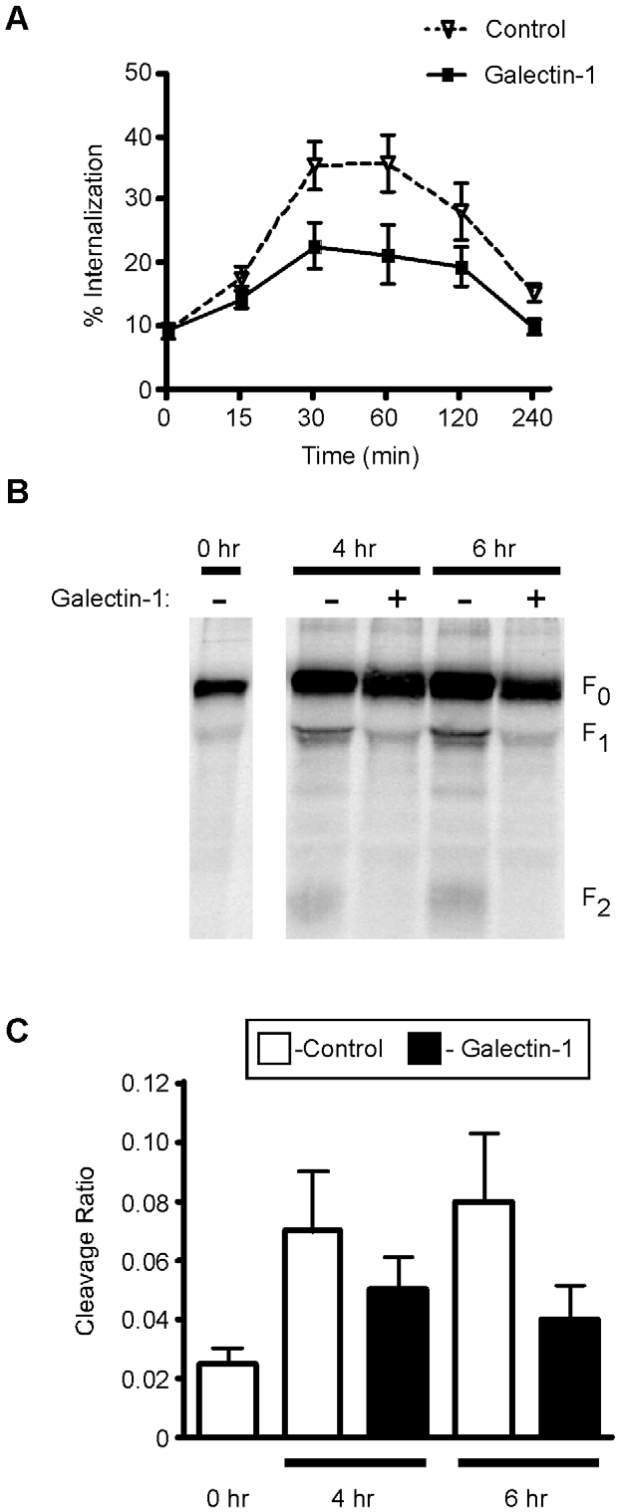
Galectin-1 inhibits NiV-F_0_ endocytosis and maturation. **A**, Galectin-1 decreases internalization of NiV-F_0_ from the plasma membrane. Cells transfected with NiV-F were cell surface biotinylated, then incubated in the presence of 20µM galectin-1 (bold line), or buffer control (dashed line), for the indicated times to allow internalization. Internalized biotinylated NiV-F was quantified by ELISA. Percent internalization was determined as the amount of internalized biotinylated NiV-F compared to total biotinylated NiV-F at the initial timepoint. Data are mean ± SEM for seven replicate experiments. **B**, Galectin-1 inhibits NiV-F_0_ proteolytic processing. 293T cells expressing NiV-F were pulse-labeled with ^35^S-methionine, then chased for 4 or 6 hrs in the presence or absence of galectin-1. NiV-F was immunoprecipitated with anti-NiV-F polyclonal sera and proteolytic processing analyzed by immunoblotting. **C**, Graphic representation of data in B. Cleavage ratio was determined as the amount of processed NiV-F (F_1_+F_2_) compared to total NiV-F protein. Data are mean ± SEM of three replicate experiments.

The formation of galectin-glycan lattices on the cell surface has also been shown to reduce the rate of endocytosis of cell surface glycoproteins [25], [26]. As described above, NiV-F is produced as an immature precursor, NiV-F_0_, which is expressed on the cell surface and undergoes endocytosis and proteolytic processing to produce the fusion competent mature protein NiV-F_1/2_. We asked if cell surface galectin-1, in addition to reducing lateral mobility of NiV-F, altered endocytosis of NiV-F_0_. Cells expressing NiV-F were biotinylated to label cell surface proteins and incubated at 37°C to allow for endocytosis in the presence or absence of galectin-1. Following endocytosis, remaining cell surface biotin was removed by reduction with glutathione, and internalized biotinylated NiV-F was quantified in cell lysates. Addition of galectin-1 decreased the amount of internalized NiV-F by approximately 50%, compared to control-treated cells (Fig. 4A). Galectin-1 also decreased the rate of NiV-F internalization by approximately 50%, (Fig. 4A) over the first 30 min, before reaching equilibrium.

As galectin-1 inhibited endocytosis of NiV-F from the cell surface, and NiV-F endocytosis is required for proteolytic processing [11], [27], we investigated the effect of galectin-1 on proteolytic processing of NiV-F. Cells expressing NiV-F were pulse labeled and chased in the presence or absence of galectin-1. As shown in Fig. 4B, galectin-1 treatment decreased the processing of NiV-F_0_ into NiV-F_1/2_. At time 0, there was little to no NiV-F_0_ cleavage, indicated by the absence of F_1_ and F_2_ bands. After four to six hrs, a fraction of NiV-F_0_ was cleaved into NiV-F_1_ and NiV-F_2_; however, the presence of galectin-1 significantly reduced processing of NiV-F_0_ as evidenced by the decrease in NiV-F_1_ and NiV-F_2_. The amount of cleavage was quantified by comparing the amount of processed NiV-F (F_1_+F_2_) to total NiV-F protein. Addition of galectin-1 decreased NiV-F processing by approximately 50% at the 6 hour time point (Fig. 4C), which is approximately the amount of endocytosis reduction seen in the presence of galectin-1 (Fig. 4A). Taken together, these results demonstrate that galectin-1 reduces NiV-F processing by binding to and decreasing internalization of the NiV-F_0_ precursor.

### Galectin-1 inhibits the function of mature NiV-F_1/2_ fusion protein

To further explore the interaction between galectin-1 and NiV-F, we used a quantitative heterologous fusion assay [13], [20], which combines ephrinB2 positive cells stably expressing the T7 polymerase (BSRT7) and ephrinB2 negative cells (PK-13) transfected with NiV-F, NiV-G and a luciferase construct with a T7 dependent promoter; in this system, luciferase expression is dependent on fusion of the two cell types. We used this assay to ask if galectin-1 inhibition of fusion involved interaction of galectin-1 with mature NiV-F, as well as inhibition of NiV-F_0_ endocytosis and maturation, as seen in Fig. 4. We found that, in this assay, addition of galectin-1 inhibited cell fusion by 80%, and galectin-1 mediated inhibition required the dimeric form of galectin-1, as a monomeric galectin-1, N-Gal-1, was not effective at blocking cell fusion, while a covalently linked galectin-1 dimer (GG) was effective at blocking fusion (Supplmentary Fig. S1A). In addition, in this assay, galectin-1 mediated inhibition of fusion was specific and carbohydrate mediated, as the inhibitory effect was abrogated by addition of lactose, a preferred glycan ligand for galectin-1, but not by sucrose (Supplementary Fig. S1B).

**Figure 5:**
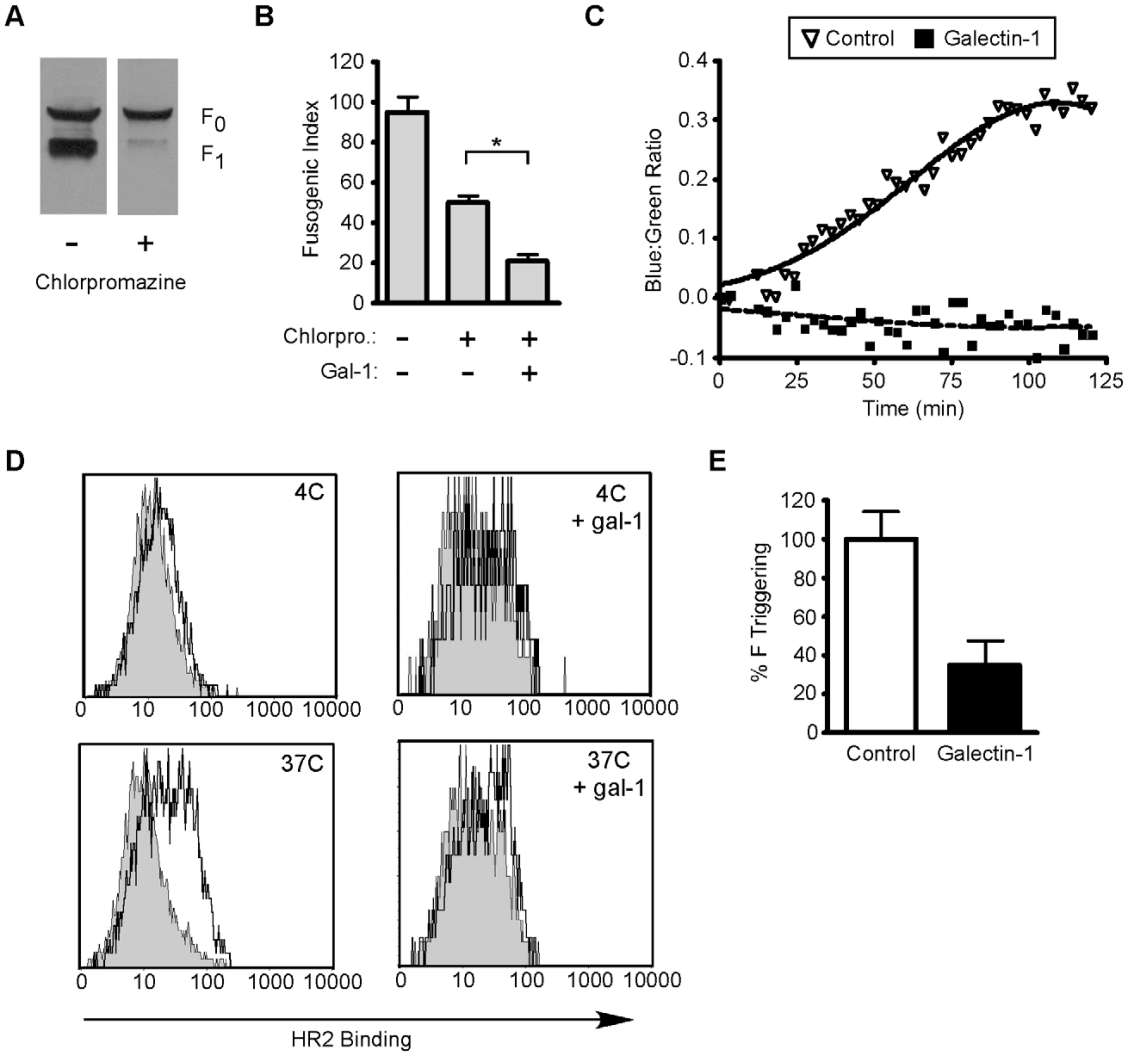
Galectin-1 inhibits function of mature NiV-F. **A**, Chlorpromazine inhibits maturation of NiV-F_0_. PK-13 were transfected with NiV-F in the absence or presence of chlorpromazine (50µM). Cells were incubated overnight and NiV-F_0_ and NiV-F_1_ detected by immunoblotting. **B**, Galectin-1 inhibits heterologous cell fusion in the presence of chlorpromazine. BSRT7 ephrinB2 positive cells were added to a monolayer of PK13 cells transfected with NiV-F, NiV-G and a luciferase construct with a T_7_ dependent promoter in the presence or absence of chlorpromazine (50µM) and galectin-1 (20µM). * p = 0.0002, calculated using Student's t test. **C**, Fusion kinetics in the presence or absence of galectin-1. NiV-G and NiV-F were expressed in effector PK13 cells, and the relative rate of fusion assessed on target 293T cells loaded with CCF2 dye. Relative fusion is the ratio of blue to green fluorescence from NiV-G and NiV-F-transfected effector cells minus the ratio of background blue to green fluorescence from empty-vector (pcDNA3)-transfected cells. Each data point is the mean of three independent experiments. **D**, Galectin-1 inhibits the ability of NiV-F to be triggered for membrane fusion. CHO cells expressing NiV-F and NiV-G were mixed with CHO cells (negative control, grey shaded) or CHOB2 cells (ephrinB2 positive, black line) for 1.5 hr at 4°C. Cell mixtures were brought to 37°C or kept at 4°C for 1.5 hr with 1 µM biotinylated HR2 peptide, in the presence or absence of galectin-1GG; top, 4°C without and with galectin-1; bottom, 37°C without and with galectin-1. **E**, Inhibition of F triggering at 37°C; data are mean fluorescence intensity of triplicate determinations ± SEM. See also Supplementary Figure S1.

Chlorpromazine is an endocytosis inhibitor that reduces NiV-F endocytosis, cleavage, and maturation [27]; cells treated with chlorpromazine at the time of transfection with NiV-G and NiV-F demonstrated virtually no maturation of nascent NiV-F_0_ (Fig. 5A). We reasoned that, if galectin-1 plus chlorpromazine were added to cells already expressing mature NiV-F, any effect of galectin-1 on cell fusion would indicate that galectin-1 bound to and directly inhibited the fusogenic activity of mature NiV-F, rather than reducing maturation of NiV-F_0_. Indeed, we found that addition of galectin-1 to chlorpromazine treated cells inhibited syncytia formation above the level of inhibition observed with chlorpromazine alone (Fig. 5B), suggesting that galectin-1 can inhibit the function of the mature fusion protein, in addition to inhibiting endocytosis of NiV-F_0_.

To further demonstrate that galectin-1's inhibitory activity can be effected through mature NiV-F, we used a real-time fusion kinetics assay [28] (Fig. 5C). Non-permissive effector cells (receptor-negative) were co-transfected with beta-lactamase, NiV-G, and NiV-F and then added to ephrinB2-expressing 293T target cells labeled with CCF2-AM dye. Cell-cell fusion was detected by analyzing the shift from green to blue fluorescence, indicating cytoplasmic mixing and beta-lactamase cleavage of CCF2-AM. In the absence of galectin-1, cell-cell fusion plateaued at about 100 min after mixing of the cells, while addition of galectin-1 completely inhibited fusion in this assay. As we start to observe an effect of galectin-1 on cell fusion within 25 min, galectin-1 is likely affecting mature fusion protein already on the effector cell surface, rather than solely affecting maturation of nascent NiV-F_0_.Thus, the ability of galectin-1 to inhibit fusion in this assay further supports a direct interaction between galectin-1 and the mature NiV-F fusion protein.

To more specifically understand the effect of galectin-1 binding to mature NiV-F on the fusion process, we examined triggering of NiV-F in the presence and absence of galectin-1. Current models of Nipah virus membrane fusion suggest that NiV-G binding to the cell surface receptor ephrinB2 triggers a conformational change in NiV-F; this triggering results in exposure of the fusion peptide in the pre-hairpin intermediate (PHI). The PHI then undergoes six-helix bundle formation, which is the conformational change that physically drives fusion of opposing membranes [29]. In NiV-F, the two heptad repeat regions, HR1 and HR2, fold next to each other during six-helix bundle formation. The HR1 region is transiently exposed during PHI formation, but before six-helix bundle formation. We have previously demonstrated [23] that a biotinylated peptide corresponding to the HR2 region can inhibit NiV-F mediated fusion by binding to the exposed HR1 region during PHI formation; HR2 peptide binding serves as a functional assay for formation of the PHI or NiV-F triggering. As Figs. 5B and 5C indicated that galectin-1 can block the fusogenic activity of mature NiV-F, we asked if galectin-1 binding to mature NiV-F could inhibit the conformational change in NiV-F (triggering or PHI formation) necessary to expose the fusion peptide, thereby inhibiting membrane fusion and syncytia formation.

To detect triggering, wild type CHO cells or CHO cells stably expressing ephrinB2 (CHOB2) were mixed with CHO cells transfected with NiV-F and G at 4°C for 1.5 hrs; NiV-G binding to ephrinB2 is an energy independent process. The biotinylated HR2 peptide was added to the cells in the presence or absence of galectin-1, and fusion was induced by incubation at 37°C for an additional 1.5 hours, because PHI formation is an energy dependent process. Binding of the HR2 peptide indicates that triggering has occurred. We observed no triggering of NiV-F at 4°C, regardless of the presence of galectin-1 (Fig. 5D). In the absence of galectin-1, incubation of the cell mixture at 37°C resulted in NiV-F triggering as seen by an increase in HR2 peptide binding. However, addition of galectin-1 reduced HR2 peptide binding, demonstrating that galectin-1 inhibited triggering of mature NiV-F (Fig. 5D). The change in HR2 peptide binding in the presence of galectin-1 is quantified in Fig. 5E. These results clearly show that galectin-1 can directly inhibit NiV-F triggering, so that this effect, in addition to inhibition of NiV-F lateral movement on the cell surface and inhibition of NiV-F_0_ maturation, contributes to the mechanism of galectin-1 mediated inhibition of NiV-F mediated cell fusion.

### Site-specific glycosylation on NiV-F

**Figure 6:**
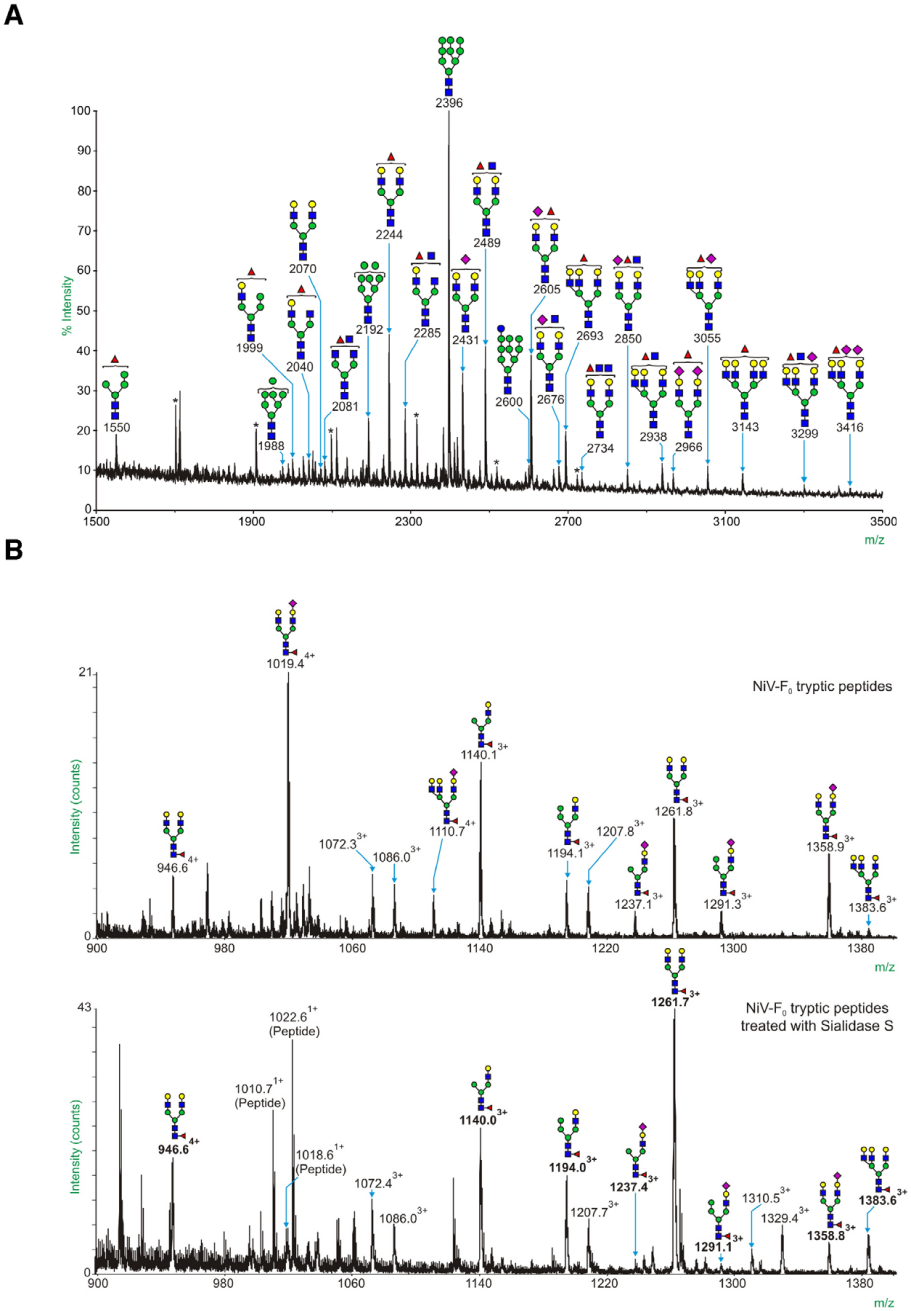
The F3 N-glycan on NiV-F is a complex N-glycan containing putative binding sites for galectin-1. **A**, MALDI-TOF mass spectrum of all permethylated N-glycans from NiV-F_0_. Annotated structures were deduced by taking into account theoretical compositions and knowledge of the biosynthetic pathways (for further information, refer to http://www.ncbi.nlm.nih.gov/books/bv.fcgi?rid=glyco.TOC&depth=2). All molecular ions are [M+Na]^+^. Peaks labeled with * represent contaminating hexose polymers. Unlabelled peaks are non-carbohydrate contaminants or permethylation products. **B**, Glycan component of the F3 glycopeptide, GALEIYK**N**NTHDLVGDVR, and effect of sialidase S digestion. *Top panel* – NiV-F_0_ was digested with trypsin and the peptide/glycopeptide mixture was analysed by LC-ES-MS/MS; the summed MS data for the F3 glycopeptide are shown. *Bottom panel* – The LC-ES-MS/MS experiment was repeated after treatment of the tryptic digest of NiV-F_0_ with Sialidase S; summed MS data for the partially desialylated F3 glycopeptide are shown. Unannotated peaks correspond to peptides. Molecular ions attributable to glycopeptides are annotated with m/z values and subscripted charge states. Peaks labeled in bold correspond to molecular ions that have shifted on Sialidase S digestion; these peaks are also assigned a potential glycan structure. Symbol nomenclature is that used by the Consortium of Functional Glycomics (CFG) (see key below). See also Figure S2. Key: Galactose (yellow circle), Mannose (green circle), GlcNAc (blue square), Fucose (red triangle), NeuAc, (purple diamond).

The NiV-F fusion protein contains 5 consensus sites for N-glycosylation, labeled F1–F5; four of these predicted sites have been found to be glycosylated *in vivo* (F2–F5) [13]. To establish the types of N-glycans expressed by NiV-F_0_ and NiV-F_1_, glycomic screening was performed using MALDI-TOF mass spectrometry. The N-glycans were released by PNGase F and analyzed as their permethylated derivatives. The resulting spectra exhibit a series of singly charged sodiated molecular ions ([M+Na]^+^) to which putative structures are assigned based on the molecular compositions and knowledge of the N-glycan biosynthetic pathway. The profile for the complete propeptide, NiV-F_0_, can be seen in Fig. 6A. The assigned structures on NiV-F_0_ include high mannose (m/z 1988, 2192, 2396; Man_7–9_GlcNAc_2_), complex (m/z 2040–3416; Fuc_0–1_NeuAc_0–2_Hex_0–7_HexNAc_4–6_) and hybrid structures (m/z 1550, 1999; Fuc_1_Hex_4–5_HexNAc_2–3_). The complex and hybrid structures contain lactosamine (Gal-GlcNAc) moieties that can be recognized by galectin-1. The MALDI-TOF mass spectrum of permethylated N-glycans from NiV-F_1_ is displayed in Supplementary Figure S2. Comparison of the structures released from NiV-F_0_ and NiV-F_1_ suggests that the glycosylation sites on the NiV-F_2_ subunit (F2 and F3 glycans) are modified with larger complex structures than those on NiV-F_1_ subunit (F4 and F5 glycans).

Previous research has shown that galectin-1 preferentially binds to the F3 glycan and this contributes significantly to galectin-1 inhibition of NiV-F mediated fusion of Vero cells. The other NiV-F N-glycans can also contribute to galectin-1 binding, as shown by co-immunoprecipitation studies with the NiV-F3 mutant [20]. In order to confirm the differences in site glycosylation suggested in the comparative MS data, and, where possible, deduce which subtypes of N-glycan occur at each glycosylation site, we performed online nano-LC ES-MS and data-dependent MS/MS analyses on tryptic peptides and glycopeptides from purified NiV-F samples. A peptide containing the F3 glycosylation site (GALEIYK**N**NTHDLVGDVR) was observed at an elution time of ∼50 min (Figure 6B, top panel). Carbohydrate structures were assigned by identifying neighboring ions separated by mass differences corresponding to sugar residues. At the F3 site, a total of 26 different glycan compositions were assigned. The carbohydrate structures are mostly complex and hybrid-type structures. The most intense peak assigned in Figure 6B (top panel) corresponds to a mono-sialylated, core-fucosylated bianntennary carbohydrate structure (m/z 1019.38^4+^). Previous research has shown that alpha 2,6-linked sialic acid caps block galectin-1 binding while alpha 2,3 sialic acid caps do not (Stowell et al. 2008). Treatment of the tryptic glycopeptides prior to the nano-LC ES-MS experiment with Sialidase S (a sialidase that specifically cleaves the alpha 2,3 linked sialic acid) revealed that both alpha 2,3 and alpha 2,6-linked sialic acid are present, with 2,3-linked sialic acid being the more abundant (Fig. 6B, lower panel). These results suggest that the F3 glycan contains abundant putative binding sites for galectin-1. Analysis of the other glycosylation sites revealed that the F5 site carries only high mannose-type glycans (Supplementary Fig. S3), indicating that the F5 glycan is unlikely to contribute substantially to galectin-1 binding. The F4 site carries mostly complex-type structures, and the major peak at F4 is the sialylated biantennary glycan without core fucose (Supplementary Fig. S4), indicating that the F4 glycan may contribute to galectin-1 binding.

### Galectin-1 inhibits NiV-F maturation and fusion through binding to the F3 glycan

**Figure 7:**
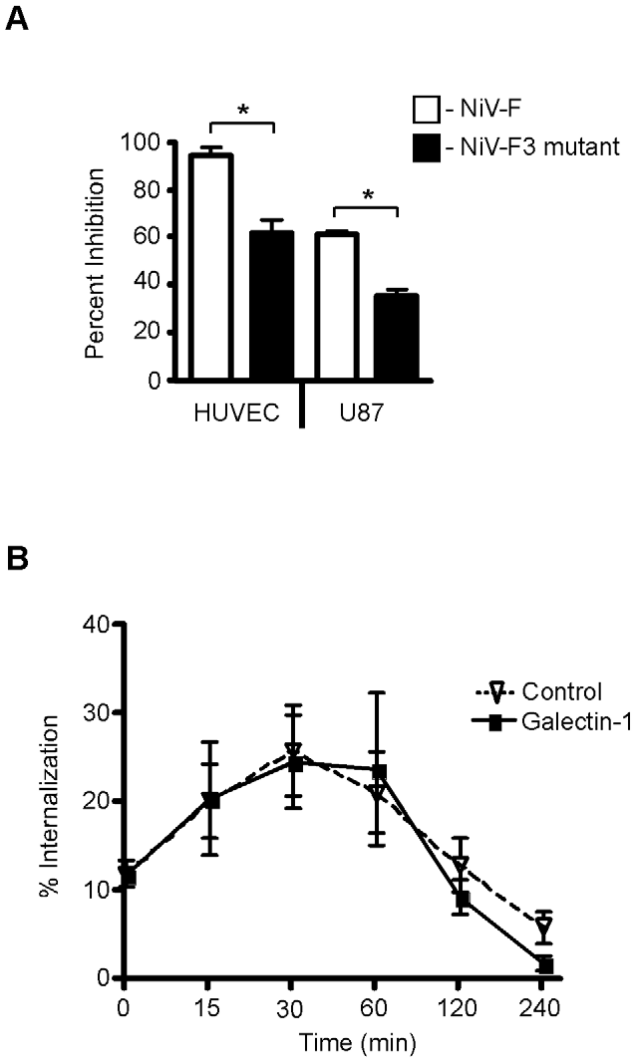
The F3 glycan is critical for galectin-1 inhibition of NiV-F maturation and function. **A**, The F3 mutant (NiV-F missing the F3 glycan) is resistant to galectin-1 inhibition of syncytia formation in endothelial and glial cells, using the heterologous cell fusion assay described in Fig. 1. PK13 cells expressing NiV-G and either wildtype NiV-F (white) or NiV-F lacking the F3 glycan (black) were added to indicated cells in the presence of galectin-1 (HUVEC, 10µM; U87, 20µM). The y-axis shows percent inhibition of fusion. Data are mean ± SD of triplicate samples from one of three replicate experiments. * p = 0.0001, calculated using unpaired Student's t test. **B**, The F3 glycan on NiV-F is critical for galectin-1 inhibition of NiV-F_0_ internalization. Cells transfected with NiV-F3 (lacking the F3 glycan) were cell surface biotinylated and incubated in the presence of galectin-1 (20µM) (bold line), or buffer control (dashed line), for the indicated times to allow internalization. Internalized NiV-F3 was quantified as in Fig. 4. Data are mean ± SEM for seven replicate experiments.

We previously found that only the F3 glycan on NiV-F, but not the other N-glycans, is critical for optimal galectin-1 binding and inhibition of fusion of Vero cells [20]. Thus, we asked if the F3 glycan was also important for galectin-1 inhibition of fusion of endothelial cells and glial cells, the target cells of NiV. PK13 cells transfected with NiV-G and NiV-F or NiV-F3, lacking the F3 glycan, were added to HUVECs and U87 cells, and syncytia formation was scored in the presence or absence of galectin-1 (Fig. 7A). There was reduced inhibition of fusion by galectin-1 between PK13 cells expressing NiV-F3 and target cells, compared to cells expressing wildtype NiV-F3. These results indicate that galectin-1 partially inhibits fusion in relevant cell types by binding to the F3 glycan on NiV-F.

We also asked if the F3 glycan on NiV-F_0_ is critical for galectin-1 inhibition of endocytosis and maturation, as seen in Fig. 4A. The reduced endocytosis of NiV-F_0_ in the presence of galectin-1 was essentially abrogated in cells expressing the NiV-F3 mutant (Fig. 7B); in addition, we quantified processed NiV-F and NiV-F3, and found that, as we saw in Fig. 4B, processing of wildtype NiV-F was reduced approximately 50% in the presence of galectin-1, while there was <20% reduction of NiV-F3 processing in the presence of galectin-1 (data not shown). Taken together these data indicate that the F3 glycan on NiV-F is critical for galectin-1 inhibition of endocytosis of immature NiV-F_0_.

## Discussion

The galectins, a family of mammalian carbohydrate binding proteins, interact with a broad range of mammalian cell surface glycoproteins to regulate essential functions in virtually every type of cell, including neural, immune and endothelial cells. Recent reports have shown that galectins can also directly bind glycoproteins on microbial pathogens, including viruses, bacteria and fungi, and that some galectins can participate as innate immune effectors. For example, galectin-3 binding to *Candida albicans* is fungicidal [30] and galectin-4 and galectin-8 binding to *Escherichia coli* is bacteriocidal [31]. Conversely, some pathogens exploit endogenous galectins to promote infection or evade host immune responses [32], [33], [34], [35], [36], [37], [38], [39]. Enveloped viruses use glycoproteins as both fusion and attachment molecules, and galectins have been shown to interact with envelope glycoproteins on HIV, HTLV and NiV [20], [33], [35], [37], [40]. Previous work from our laboratories demonstrated that both NiV-G and NiV-F glycoproteins bind galectin-1, and that exogenous galectin-1 reduced syncytia formation triggered by NiV-F; galectin-1 inhibition of syncytia formation was specific for paramyxoviruses, as we did not observe inhibition of cell fusion induced by HTLV-2, vaccinia and MLV [20]. As inflammation and viral infection can stimulate production of galectins by endothelial cells [15], [16], [18], endogenous galectin-1 may contribute to host defense against NiV infection by mitigating the endothelial cell syncytia formation that is a hallmark of Nipah infection, as we have clearly demonstrated that endogenous galectin-1 can attenuate endothelial cell fusion *in vitro* (Fig. 2). While autopsy studies of patients who succumbed to NiV infection found endothelial cell syncytia in numerous organs including brain [2], [3], variation in galectin-1 expression among infected individuals may contribute to susceptibility or resistance to viral-induced pathophysiology and, in part, explain why some infected individuals do not progress to encephalitis.

We investigated the effect of galectin-1 at each stage of the cell fusion process, and have identified three points at which galectin-1 inhibits NiV-F mediated syncytia formation. First, galectin-1 reduced lateral mobility of NiV-F on the plasma membrane (Fig. 3). Second, galectin-1 retarded endocytosis and maturation of the precursor NiV-F_0_ (Fig. 4). Third, galectin-1 prevented the triggering of mature NiV-F into the fusion-competent form (Fig. 5). Lateral mobility in the plasma membrane is important for NiV-F mediated cell fusion, as the physical separation of NiV-F and NiV-G is required for the conformational change in NiV-F necessary for membrane fusion [23], [28]. Galectin-1 inhibition of the lateral movement of NiV-F could contribute to reduced ability of mature NiV-F to form the fusion competent PHI on the cell surface, in addition to the direct inhibition of PHI formation that we observed in Fig. 5.

All these effects are congruent with data from prior studies demonstrating that galectin interacts with endogenous mammalian plasma membrane glycoproteins to form galectin-glycoprotein lattices on the cell surface [22], [41]. Formation of these lattices increases the local concentration of galectins at the cell surface, and also has direct effects on the glycoproteins in the lattice, as galectin-glycoprotein lattices can regulate the distribution, residence time, and function of glycoproteins on the plasma membrane [24], [25], [42], [43], [44], [45]. Galectin-glycoprotein lattice formation has been shown to inhibit lateral diffusion of EGF receptors on tumor cells [24]. Galectin-glycoprotein lattices also reduce endocytosis of EGF receptors on tumor cells, Glut-2 receptors on pancreatic cells, and receptor tyrosine phosphatase beta on neural cells [24], [25], [26]. The present study provides the first demonstration that galectin-1 on the cell surface reduces lateral mobility and endocytosis of a viral glycoprotein, rather than an endogenous mammalian glycoprotein. Lattice formation between galectin-1 and NiV-F likely contributes to inhibition of NiV-F maturation, reduced mobility, and reduced triggering that we observed.

Our findings also emphasize the specificity of the interaction between galectin-1 and unique glycans on a viral envelope glycoprotein. Glycoproteomic analysis showed that the most abundant glycan at the F3 site is a monosialylated biantennary glycan with a 2–3 linked sialic acid (Fig. 6). This structure fulfils the known requirement for a galectin-1 ligand [46]. Fig. 7, as well as our previous report, shows that the F3 glycan on NiV-F participates in galectin-1 inhibition of syncytia formation at two distinct points; the F3 glycan appears essential for galectin-1 inhibiting maturation of NiV-F_0_ and also substantially contributes to the galectin-1 effect on the function of mature NiV-F. This level of specificity for a particular glycan on a glycoprotein counter-receptor is quite novel, in that previous reports on mammalian lectins interacting with viral glycoproteins emphasized simple pattern matching or binding to dense and repetitive arrays of terminal monosaccharide ligands [47], [48], [49]. In contrast, a significant component of the interaction between galectin-1 and NiV-F primarily involves a single glycosylation site on the viral glycoprotein. This interaction emphasizes the site-specific nature of glycan addition and subsequent modification during viral glycoprotein maturation and transport to the cell surface. As shown in Fig. 6, there is significant heterogeneity among the different glycans attached to NiV-F. Similar microheterogeneity has been observed for the glycans attached to HIV gp120 [50]. This microheterogeneity would substantially contribute to selective interaction of viral glycoproteins with endogenous mammalian lectins, such as C-type lectins and galectins, or with antibodies that recognize specific glycans on viral glycoproteins. Interestingly, F3 glycan removal resulted in the highest levels of hyperfusiogenicity compared to the removal of other glycans on NiV-F [13]. This is consistent with F3 glycan removal reducing the inhibitory effects of endogenous galectin-1.

This report defines several molecular mechanisms by which galectin-1 binding to NiV-F interferes with maturation and function of NiV-F to reduce cell fusion, and also demonstrates that endogenous endothelial galectin-1 can influence the extent of syncytia formation. Since syncytia formation is the pathognomonic hallmark of NiV infection, we predict that, *in vivo*, galectin-1 may reduce pathophysiologic consequences during the course of an infection. An 11kb region containing the gene coding for galectin-1 contains 14 different single nucleotide polymorphisms (SNP) [51], and this genetic variability may partially account for the range of pathophysiological consequences seen in Nipah virus infection, as SNPs in other galectins have been found to be associated with increased disease risk [52]. Our studies focus on cell-cell fusion, as syncytia formation is the event that primarily contributes to the endothelial destruction and hemorrhagic sequelae in NiV infection. However, galectins may also influence attachment of viruses to target cells [33], [35], [37], [40], although galectin mediated cell attachment is fundamentally distinct from the specific fusion inhibitory mechanisms that we have elucidated in this study. Galectins can play multiple distinct roles in complex biologic processes such as pathogen entry, replication and dissemination, so that our goal is to define all the roles played by galectin-1 during the entire course of Nipah virus infection of human host cells.

## Materials and Methods

### Cell lines and reagents

Vero cells and CHO cells (ATCC) were maintained in MEM alpha (Invitrogen) with 10% FBS (Hyclone) and 2mM Glutamax in 5%CO_2_ at 37°C. PK-13 porcine fibroblast cells and 293T cells were maintained in DMEM (Invitrogen) with 10% FBS (Hyclone) and 2mM Glutamax. BSR cells stably transfected with T7 polymerase were maintained in DMEM with 10% FBS (Hyclone), 2mM Glutamax, and 0.5mg/ml G418 (Sigma). U87 glioblastoma cells (gift of P. Mischel, UCLA) were maintained in DMEM with 10% FBS (heat inactivated at 55°C for 30 min), 2mM Glutamax, and 50 units/ml penicillin/streptomycin. mVECs [6] and HUVECs (BD Biosciences) were maintained in MDCB-131 Complete media with fetal bovine serum and antibiotics (VEC Technologies, INC.).

Codon-optimized NiV-F and G plasmids tagged with AU1 were previously described [20]. NiV-F3 plasmid (encoding NIV-F lacking the F3 glycan) was previously described [13]. NiV-F-GFP plasmid was created by synthesizing a fusion gene between NiV-F and GFP using overlapping PCR. Oligonucleotide sequences were designed that flank the 5′ region of codon-optimized NiV-F and 3′region of GFP. An additional oligonucleotide that overlaps the 3′ region of NiV-F and the 5′ region of GFP was also designed which did not contain the stop codon at the end of the NiV-F ORF and included a GGG linker between the two genes. NiV-F (1.6 kb) and GFP (0.8kb) genes were amplified by PCR using the appropriate 5′ or 3′ oligonucleotide primers and the overlapping primer. The two PCR products were gel purified and used together as template for another PCR reaction using the original 5′ and 3′ primers. The resulting 2.2kb fusion gene product was subcloned into pcDNA3 (Invitrogen) and sequence verified. Recombinant human galectin-1 was expressed in *E. coli* and purified by affinity chromatography on lactosyl-Sepharose, as in [53]; in all assays, the buffer control includes 8mM dithiothreitol (DTT), as galectin-1 is prepared and stored in PBS with DTT.

### Heterologous fusion assay

PK-13 cells were transfected with codon-optimized, AU1-tagged NiV-F, NiV-F3, or NiV-F_GFP_ and HA-tagged NiV-G at 15ug per plasmid using Lipofectamine 2000 (Invitrogen). Cells were cultured overnight, lifted with 5mM EDTA (ethylenediaminetetraacetic acid) and overlayed in the absence or presence of galectin-1 onto ephrinB2 positive cells (Vero, U87, mVEC, or HUVEC). After 2 hrs (Vero/U87) or 6 hrs (HUVEC/mVEC), cells were fixed with 2% paraformaldehyde (EMS). After 4′,6′-diamidino-2-phenylindole (DAPI) staining (Invitrogen), nuclei inside syncytia per ×100 field were counted by fluorescence microscopy as previously described [20].

### Cell surface staining for galectin-1

HUVEC cells were incubated with or without 20µM galectin-1 for 30 min at 37°C. Cells were fixed in DTSSP (Thermo Scientific) at 0.2mg/ml for 10 min at room temperature, and quenched by addition of 100µl of 1M Tris pH 7.5 for 15 min at room temperature. Cells were washed with PBS and lifted with 5mM EDTA at 37°C for 10 min. Cells were stained with a rabbit galectin-1 antibody (Strategic) for 1 hr at 4°C. Cells were washed with PBS and stained with FITC-conjugated AffiniPure goat anti-rabbit IgG (H+L) antibody (Jackson ImmunoResearch) at 8µg/ml for 1 hour at 4°C. Cells were washed in PBS and resuspended in PBS with 1% BSA (Gemini BioProducts) for analysis by flow cytometry. Flow cytometric analysis of HUVEC cell surface galectin-1 was performed on a Becton-Dickinson FACScan, using CellQuest software (Becton-Dickinson).

### Galectin-1 knockdown

3 different siRNA constructs directed toward human galectin-1 mRNA in lentivectors (pSIH1-H1-copGFP plasmid) (System Biosciences) were packaged into VSV using pPACKH1 Lentivector Packaging Kit (System Bioscience). Lentivirus was produced in 293T cells; three different viruses corresponding to the 3 different siRNAs were isolated, as well as a virus containing only the GFP infection marker, and a virus containing siRNA to an irrelevant protein, CD43. HUVECs were plated at 1.4×10^5^ cells per well in a 6 well tissue culture dish (Corning). A single well of HUVECs was treated with each virus (MOI of 3.3, total MOI of 10) in PBS with 1% heat inactivated FBS (Hyclone) and cells were spinoculated at 2000 rpm at 37°C for 2 hrs. Infected cells were grown in full media for 4 days and knockdown was assessed by western blot and flow cytometry.

### FRAP

Fluorescence Recovery After Photobleaching analysis of Vero cells transfected with NiV-F_GFP_ plasmid was performed in 35mm glass bottom culture dishes (MatTek) on a 37°C heated stage. Cells were treated with 20µM galectin-1 or buffer control for 10 min prior to photobleaching. Images were acquired on a confocal microscope (Leica SP2 1P-FCS) with a HCX PL APO 63.0×1.40 oil objective and fully opened pinhole. Photobleaching of NiV-F-GFP was performed using 5 scans with the 488-nm laser at full power. Recovery data (six cells per time point from each of two independent experiments) was collected every 2.2 seconds for a total of 25 time points. Images were acquired with equivalent acquisition settings including pre-bleach, bleach, and post-bleach measurements. Bleaching and recovery were measured in a fixed area and compared to an area absent of fluorescence outside of the cell.

### NiV-F endocytosis assay

PK-13 cells were transfected with NiV-F or NiV-F3. After overnight incubation, cells were washed twice with KRPH buffer (128mM NaCl, 4.7mM KCl, 1.25mM CaCl_2_, 1.25mM MgSO_4_, 5mM Na_2_HPO_4_, 20mM Hepes pH 7.4) and cell surface biotinylated using the EZ-Link sulfo-NHS-SS-Biotin (Pierce). Biotinylation was quenched in 20mM glycine and cells were washed again in KRPH buffer. Cells were incubated in media with or without galectin-1 or buffer control for the designated times at 37°C to allow endocytosis. After timepoints, cells were washed and remaining biotin cleaved twice with cleavage buffer (90mM NaCl, 1.25mM CaCl_2_, 1.25mM MgSO_4_, 2mg/ml BSA, 50mM glutathione pH 8.6) and quenched in 20mM glycine for 15 min. Cells were lysed in lysis buffer (20mM Tris-HCL pH 7.5, 100mM NH_2_SO_4_, 0.1% BSA, 0.75% Trition X-100, 0.01% NaN_3_). Biotinylated NiV-F in cell lysates was quantified by ELISA using mouse anti-AU1 (1∶1000) coated Reacti-Bind goat anti-mouse plates (Pierce) to capture AU1-tagged NiV-F, and detected with streptavidin-HRP (Biorad).

### Metabolic labeling and pulse chase

293T cells grown in 6-well plates were transfected with 0.25µg of NiV-F plasmid DNA and 1.75µg pcDNA3 per well. 24 hrs post transfection, cells were incubated in media lacking methionine and cysteine for 45 min followed by labeling with media containing ^35^S-cysteine and ^35^S-methionine (100mCi/ml) for 30 min, and then in chase (non-radioactive) media for 4 to 6 hrs, in the presence or absence of 20µM galectin-1. Cells were lysed in 200µl RIPA buffer (20mM Tris-HCl pH 7.4, 137mM NaCl, 0.1% SDS, 0.5% deoxycholate, 1% NP-40, 2mM EDTA). NiV-F was immunoprecipitated from cleared cell lysates using a combination of anti-NiV-F polysera [13] and anti-AU1 antibody each at 1∶100 dilution, and Protein G agarose (Pierce). Precipitates were washed twice in RIPA buffer and twice with RIPA buffer plus 0.5M NaCl. Samples were separated on a 14% polyacrylamide gel and data were quantified and analyzed using a phosphoimager (Molecular Dynamics 445SI) and ImageQuant (v5.2).

### Fusion reporter assay

Fusion-nonpermissive PK-13 target cells were transfected with codon optimized NiV-F, NiV-G, and a plasmid containing a T7 promoter driven luciferase at a 3∶3∶1 ratio with 30µg of total plasmid DNA and grown overnight. BSR cells stably transfected with a T7 polymerase (BSRT7) were lifted with 5mM EDTA at 37°C for 10 min. BSRT7 cells were co-cultured with transfected PK-13 cells for 6 hrs with or without galectin-1. After 6 hrs, the cells were lysed in 0.3% Triton-X 100 by two rounds of freeze/thaw at −80°C. Luciferase expression was quantified using a Luciferase assay system (Promega). Briefly, lysates were transferred to a 96 well opaque black plate, luciferase assay substrate was added, and light production was measured by luminometry (Turner Biosystems).

### Chlorpromazine treatment

PK-13 cells were transfected with NiV-F and simultaneously treated with chlorpromazine (Sigma) for 16 hrs. Cells were lysed in 50mM Tris-HCl (pH 7.4), 1% Nonidet P-40, 5mM EDTA, 150mM NaCl, 1mM PMSF, 10mg/ml aprotinin, 10mg/ml leupeptin, and 10mM sodium orthovanadate with scraping. Lysates were microfuged for 15 min at 10,000 rpm. Samples were denatured in NuPAGE reducing agent and NuPAGE Sample Buffer (Invitrogen) before loading. Lysates (10 µg) were separated on a 12% Bis-Tris gel (Invitrogen NuPAGE Electrophoresis System) and electroblotted onto nitrocellulose (Whatman). The membrane was blocked and probed as previously described [53] using an AU1 antibody (Covance) and NiV-F proteins were visualized by ECL.

### Triggering of NiV-F by NiV-G in the presence or absence of galectin-1

NiV-F triggering was measured essentially as in [23] except in the presence or absence of 0.4 µM galectin-1GG [54]. Briefly, CHO cells were transfected with NIV-F and NiV-G expression plasmids, plus GFP expression plasmid, at a 13∶6∶1 ratio, respectively. 18 hrs post-transfection, a 1∶1 ratio of transfected cells and either CHO (negative control) or CHOB2 (CHO cells transfected with ephrinB2 [23]) cells were mixed and incubated for 2 hr at 4°C, followed by a 90 min incubation at either 4°C or 37°C in the presence of excess biotinylated HR2 peptide and in the presence or absence of 0.4 µM galectin-1GG, as indicated. Cells were washed with wash buffer (1% FBS in PBS), fixed in 0.5% paraformaldehyde in wash buffer, and washed twice with wash buffer. Biotinylated HR2 peptide bound to F was detected using streptavidin-APC (ebioscience). GFP-positive cells were gated and analyzed for HR2-biotin binding.

### Fusion kinetics in the presence or absence of galectin-1

Fusion kinetics were determined in a beta-lactamase reporter cell-cell fusion assay, as previously described [13], [28], [55], [56], using a catalytically enhanced and codon optimized beta-lactamase gene [57], [58]. Fusion-nonpermissive PK13 effector cells were co-transfected with beta-lactamase, NiV-G, and NiV-F expression plasmids, and mixed with 293T target cells labeled with CCF2-AM dye for 30 min at 4°C. Galectin-1GG (0.4 µM) or buffer control was added and the cells moved immediately to 37°C. Cell-cell fusion was detected by analyzing the shift from green to blue fluorescence, indicating beta-lactamase cleavage of CCF2-AM. Fluorescence was quantified every 3 min with a Synergy 2 Multi-mode microplate reader (BioTek Instruments, Winooski, VT). Results are expressed as the ratio of blue to green fluorescence obtained with NiV-G- and NiV-F-transfected effectors minus the background blue to green fluorescence ratios obtained with NiV-G- and empty-vector-transfected cells.

### Preparation of NiV-F sample for mass spectrometric analysis

Glycan analysis was performed on NiV-F protein pseudotyped onto VSV viral-like particles produced in 293T cells. Gel bands containing purified NiV-F_0_ and NiV-F_1_ were destained using 100% acetonitrile, incubated with 10mM DTT for 30 min at 56°C, followed by 55 mM iodoacetic acid for 30 min at RT. Reduced and carboxymethylated NiV-F was digested with 0.5µg sequencing grade-modified trypsin (Promega, UK) at 37°C for 14 hrs. Tryptic peptides and glycopeptides were extracted from the gel pieces by incubating with 0.1% trifluoroacetic acid (TFA) and 100% acetonitrile; the supernatant was pooled and reduced in volume on a rotary evaporator. For glycomic screening the supernatant was lyophilised before being dissolved in 200µl ammonium bicarbonate (50mM, pH 8.4) and incubated with 5U of Peptide-N-glycosidase F (PNGase F) (Roche Applied Science, UK) for 24 hrs at 37°C to release the N-glycans. The reaction was terminated by lyophilisation. Glycans were separated from peptides by C_18_ Sep-Pak purification and permethylated as previously described [59].

### MALDI-TOF and online nano-LC-ES-MS/MS analysis

MALDI-TOF MS and MS/MS data on permethylated N-glycan samples were acquired in positive ion mode [M+Na]^+^ using a 4800 MALDI-TOF/TOF (Applied Biosystems, UK) mass spectrometer as previously described [60]. The MALDI data were processed using Data Explorer 4.9 Software (Applied Biosystems, UK). Tryptic digests were analysed by nano-LC-ES-MS/MS using a reverse-phase nano-HPLC system (Dionex (UK) Ltd, Camberley) connected to a quadrupole TOF mass spectrometer (API Q-STAR Pulsar I, Applied Biosystems, UK) as previously described [61]. Analysis of the ES-MS and MS/MS data was aided by use of the Peptoonist algorithm [62].

### Sialidase S digestion of NiV-F glycopeptides

Sample was dried, resuspended in 50µl of 50mM ammonium acetate (pH 5.5) and incubated with 10mU of Sialidase S at 37°C for 14 hrs. After digestion, NiV-F peptides and desialylated glycopeptides were desalted and separated using a C18-microtrap peptide cartridge (Presearch, Basingstoke). The sample was loaded directly onto the microtrap cartridge with a 25µl gastight syringe. The microtrap was first solvated with methanol, washed off with acetonitrile and conditioned with 0.1% TFA. The sample was loaded onto the column and washed with 0.1% TFA prior to eluting with 15µl 30 and 60% acetonitrile in 0.1% TFA, respectively. Eluted fractions were combined and dried down gently under nitrogen before online LC-ES-MS and MS/MS analysis.

## Supporting Information

Figure S1 Heterologous fusion is inhibited by galectin-1. Galectin-1 inhibits function of the mature fusion protein. **A**, Galectin-1 inhibits heterologous fusion in a dimer dependent manner. EphrinB2 positive cells stably expressing the T_7_ polymerase were added to a monolayer of ephrinB2 negative cells transfected with NiV-F, NiV-G and a luciferase construct with a T_7_ dependent promoter. Luciferase expression correlates with cell fusion. Data are shown as percent fusion based on the total fusion without any treatment (Lane 2). Lane 1 is a negative control and lane 3 is heterologous fusion with buffer alone. Lane 4, 30µM galectin-1. Lane 5, 10µM forced galectin-1 dimer (GG). Lane 6, 30µM monomeric galectin-1 mutant, N-Gal-1. **B**, Galectin inhibits heterologous fusion in a carbohydrate dependent manner. The galectin-1 effect of fusion inhibition was abrogated by addition of the cognate disaccharide lactose, but not by sucrose. In both panels, data are the mean + S.D. of a representative experiment performed in triplicate.
(0.05 MB TIF)

Figure S2 MALDI-TOF mass spectrum of permethylated N-glycans from NiV-F_1_. Data were acquired in the positive ion mode and all molecular ions are [M+Na]^+^. Peaks labeled with * represent contaminating hexose polymers. Peak assignments are based on theoretical compositions together with knowledge of the biosynthetic pathways. Symbol nomenclature is that used employed by the Consortium for Functional Glycomics (CFG) for the representation of glycan structures. Key: Galactose (yellow circle), Mannose (green circle), GlcNAc (blue square), Fucose (red triangle), NeuAc, (purple diamond).
(0.32 MB TIF)

Figure S3 The structure of the glycans found at the F5 glycosylation site. The table shows the glycan compositions of the glycopeptide, VDISSQISSM**N**QSLQQSK, observed as doubly, triply and quadruply charged molecular ion signals in the MS data summed between the ion retention times 51.8–52.9 min (data not shown). The m/z values in the table correspond to the smallest isotope in each cluster and the Mr values are calculated accordingly. Potential structures of the glycan compositions are given, deduced by taking into account prior glycomic experimental data and knowledge of the biosynthetic pathways. Key: Galactose (yellow circle), Mannose (green circle), GlcNAc (blue square), Fucose (red triangle), NeuAc, (purple diamond).
(0.65 MB TIF)

Figure S4 The structure of the glycans found at glycosylation site, F4. The glycan compositions of the glycopeptide, AISQSGTLLMID**N**TTCPTAVLGNVIISLGK, observed as doubly, triply and quadruply charged molecular ion signals in the MS data summed between the ion retention times 84.7–86.8 min (data not shown). Key: Galactose (yellow circle), Mannose (green circle), GlcNAc (blue square), Fucose (red triangle), NeuAc, (purple diamond).
(0.81 MB TIF)
