# Supplementary figures and images for: Endothelial Galectin-1 Binds to Specific Glycans on Nipah Virus Fusion Protein and Inhibits Maturation, Mobility, and Function to Block Syncytia Formation
Source: PLoS Pathog. 2010 Jul 15;6(7):e1000993. doi: 10.1371/journal.ppat.1000993 (PMC2904771; doi:10.1371/journal.ppat.1000993)

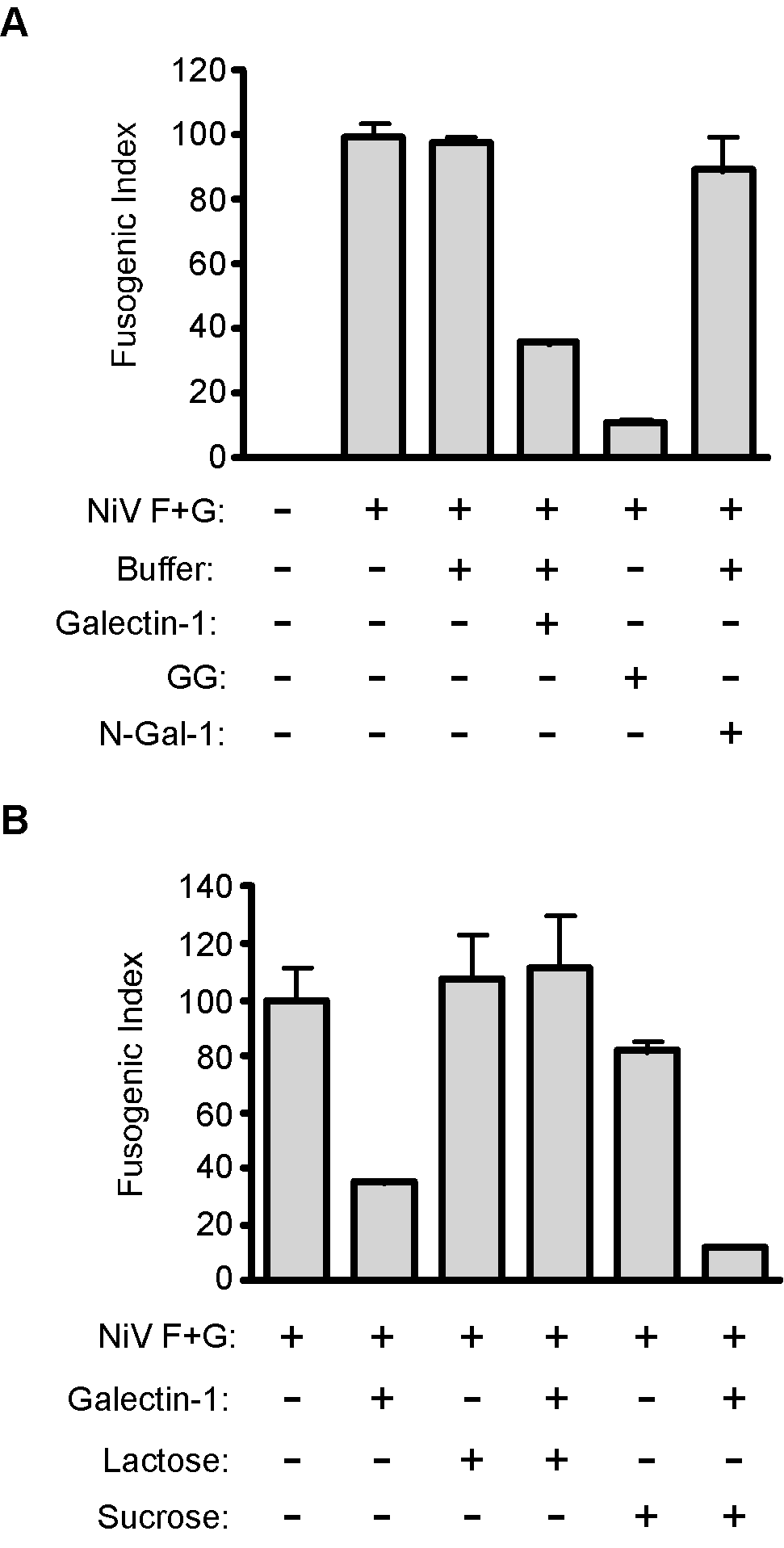

Supplement: Figure S1 — Heterologous fusion is inhibited by galectin-1. Galectin-1 inhibits function of the mature fusion protein. A, Galectin-1 inhibits heterologous fusion in a dimer dependent manner. EphrinB2 positive cells stably expressing the T7 polymerase were added to a monolayer of ephrinB2 negative cells transfected with NiV-F, NiV-G and a luciferase construct with a T7 dependent promoter. Luciferase expression correlates with cell fusion. Data are shown as percent fusion based on the total fusion without any treatment (Lane 2). Lane 1 is a negative control and lane 3 is heterologous fusion with buffer alone. Lane 4, 30µM galectin-1. Lane 5, 10µM forced galectin-1 dimer (GG). Lane 6, 30µM monomeric galectin-1 mutant, N-Gal-1. B, Galectin inhibits heterologous fusion in a carbohydrate dependent manner. The galectin-1 effect of fusion inhibition was abrogated by addition of the cognate disaccharide lactose, but not by sucrose. In both panels, data are the mean + S.D. of a representative experiment performed in triplicate. (0.05 MB TIF) [file ppat.1000993.s001.tif]

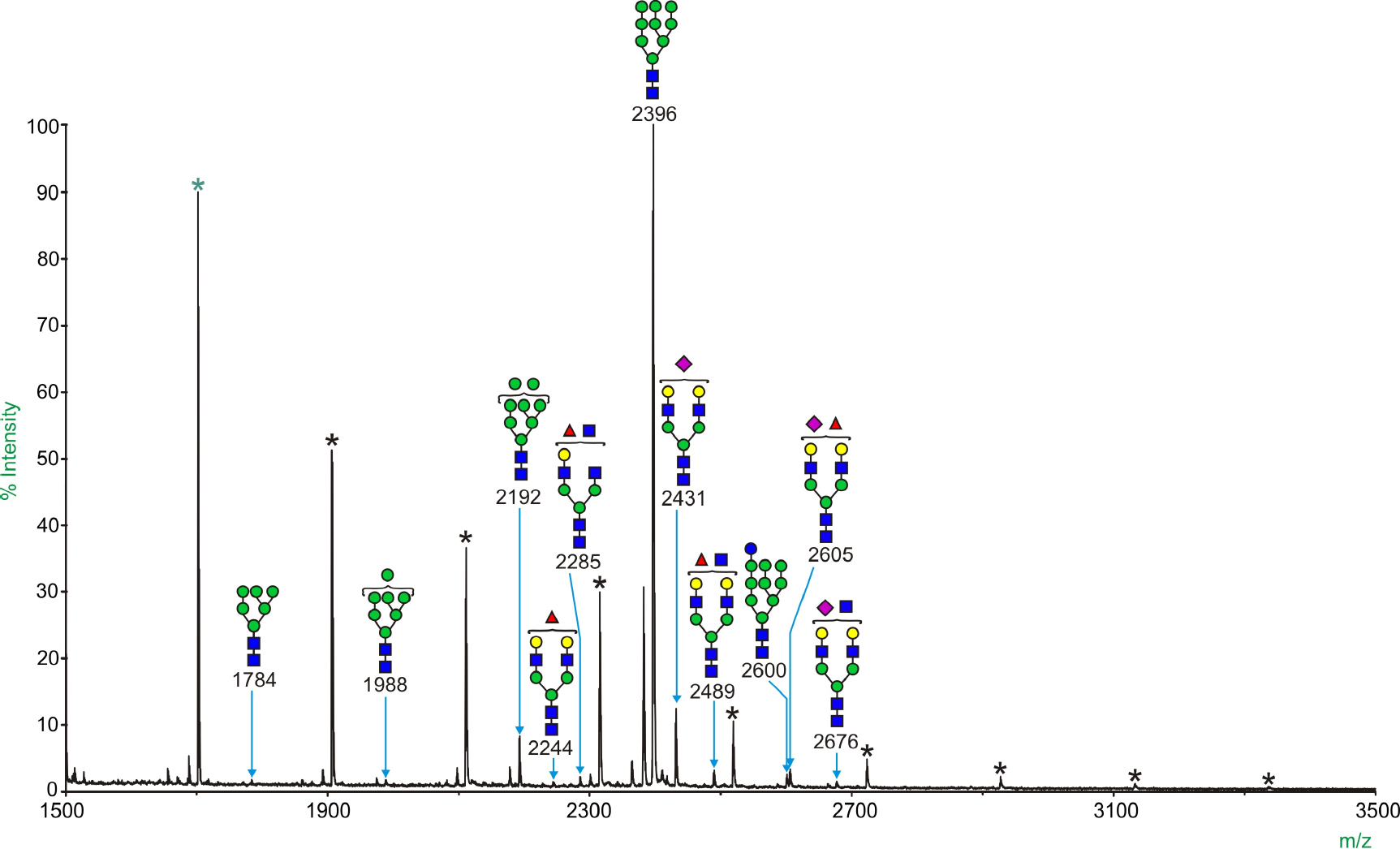

Supplement: Figure S2 — MALDI-TOF mass spectrum of permethylated N-glycans from NiV-F1. Data were acquired in the positive ion mode and all molecular ions are [M+Na]+. Peaks labeled with * represent contaminating hexose polymers. Peak assignments are based on theoretical compositions together with knowledge of the biosynthetic pathways. Symbol nomenclature is that used employed by the Consortium for Functional Glycomics (CFG) for the representation of glycan structures. Key: Galactose (yellow circle), Mannose (green circle), GlcNAc (blue square), Fucose (red triangle), NeuAc, (purple diamond). (0.32 MB TIF) [file ppat.1000993.s002.tif]

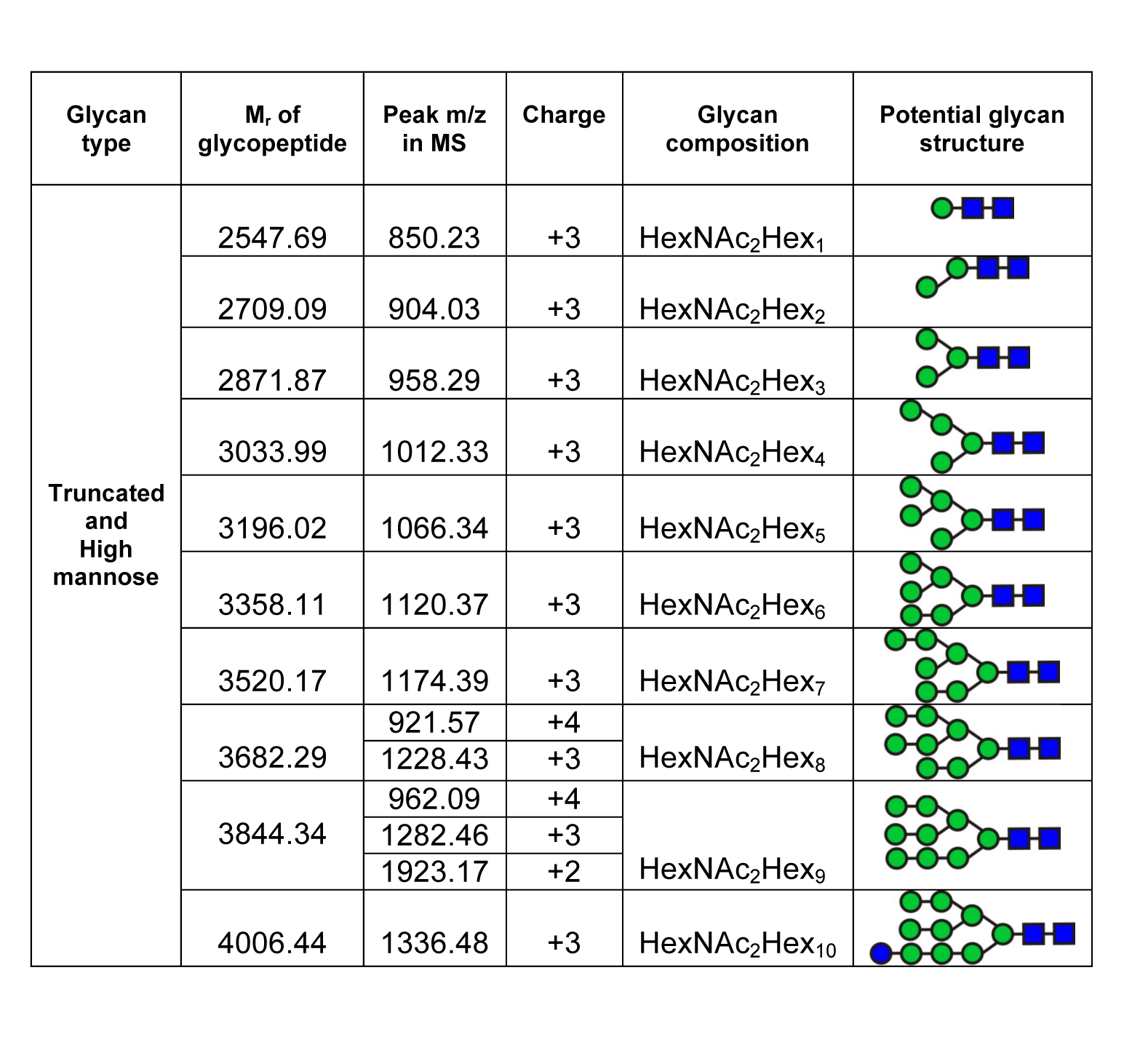

Supplement: Figure S3 — The structure of the glycans found at the F5 glycosylation site. The table shows the glycan compositions of the glycopeptide, VDISSQISSMNQSLQQSK, observed as doubly, triply and quadruply charged molecular ion signals in the MS data summed between the ion retention times 51.8–52.9 min (data not shown). The m/z values in the table correspond to the smallest isotope in each cluster and the Mr values are calculated accordingly. Potential structures of the glycan compositions are given, deduced by taking into account prior glycomic experimental data and knowledge of the biosynthetic pathways. Key: Galactose (yellow circle), Mannose (green circle), GlcNAc (blue square), Fucose (red triangle), NeuAc, (purple diamond). (0.65 MB TIF) [file ppat.1000993.s003.tif]

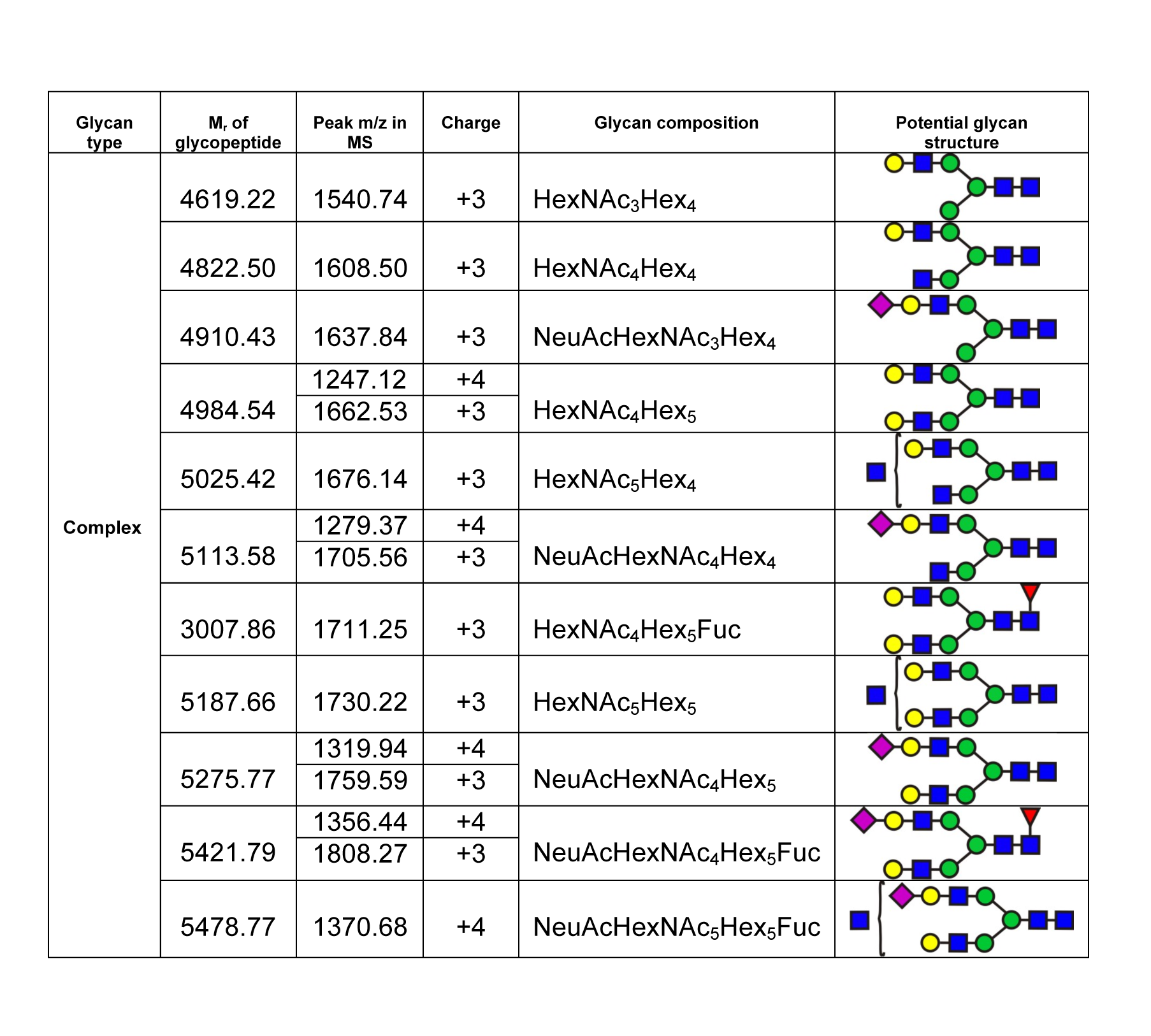

Supplement: Figure S4 — The structure of the glycans found at glycosylation site, F4. The glycan compositions of the glycopeptide, AISQSGTLLMIDNTTCPTAVLGNVIISLGK, observed as doubly, triply and quadruply charged molecular ion signals in the MS data summed between the ion retention times 84.7–86.8 min (data not shown). Key: Galactose (yellow circle), Mannose (green circle), GlcNAc (blue square), Fucose (red triangle), NeuAc, (purple diamond). (0.81 MB TIF) [file ppat.1000993.s004.tif]
